# Sensitivity of Spiking Neural Networks Due to Input Perturbation

**DOI:** 10.3390/brainsci14111149

**Published:** 2024-11-16

**Authors:** Haoran Zhu, Xiaoqin Zeng, Yang Zou, Jinfeng Zhou

**Affiliations:** College of Computer Science and Software Engineering, Hohai University, Nanjing 211100, China; zhuhr@hhu.edu.cn (H.Z.); yzou@hhu.edu.cn (Y.Z.); zhoujinfeng@hhu.edu.cn (J.Z.)

**Keywords:** spiking neural network, sensitivity, temporal coding, leaky integrate-and-fire

## Abstract

**Background:** To investigate the behavior of spiking neural networks (SNNs), the sensitivity of input perturbation serves as an effective metric for assessing the influence on the network output. However, existing methods fall short in evaluating the sensitivity of SNNs featuring biologically plausible leaky integrate-and-fire (LIF) neurons due to the intricate neuronal dynamics during the feedforward process. **Methods:** This paper first defines the sensitivity of a temporal-coded spiking neuron (SN) as the deviation between the perturbed and unperturbed output under a given input perturbation with respect to overall inputs. Then, the sensitivity algorithm of an entire SNN is derived iteratively from the sensitivity of each individual neuron. Instead of using the actual firing time, the desired firing time is employed to derive a more precise analytical expression of the sensitivity. Moreover, the expectation of the membrane potential difference is utilized to quantify the magnitude of the input deviation. **Results/Conclusions:** The theoretical results achieved with the proposed algorithm are in reasonable agreement with the simulation results obtained with extensive input data. The sensitivity also varies monotonically with changes in other parameters, except for the number of time steps, providing valuable insights for choosing appropriate values to construct the network. Nevertheless, the sensitivity exhibits a piecewise decreasing tendency with respect to the number of time steps, with the length and starting point of each piece contingent upon the specific parameter values of the neuron.

## 1. Introduction

Biological research has demonstrated that the transmission of information between neurons in the brain occurs through a temporal spike signal [1,2]. SNNs are modeled after the behavior of biological neurons [3]. The dynamical model of SNNs states that neurons integrate membrane potential by receiving inputs and emit a spike once the membrane potential reaches the threshold. In comparison to traditional neural networks, SNNs can more accurately replicate the intrinsic activities of neurons, depict the complex dynamical mechanisms of biological neurons, and integrate the information in various dimensions such as time and frequency [4,5,6,7]. The spatio-temporal dynamical characteristics and event-driven advantages of SNNs make them a promising approach for simulating the functions of the human brain [8]. Although many studies have demonstrated the biologically plausibility and low energy consumption of SNNs [9,10], the complex input–output mapping of SNNs poses challenges in quantitatively analyzing their working mechanisms and behaviors, especially in terms of the output deviations caused by the noisy inputs.

The sensitivity of neural network output to parameter perturbation enables an exploration of the network behavior, including the assessment of the error tolerance and generalization ability [11,12]. Over the past few decades, extensive research has been conducted on algorithms and applications related to the output sensitivity of neural networks. In the field of multilayer perceptron (MLP), Zeng and Yeung [13] defined the sensitivity as the output deviation caused by the input and weight perturbations and developed a generalized approach to quantify the sensitivity of both ensemble MLPs and individual MLPs. Yeung and Sun [14] extended the existing method for sensitivity derivation by removing the restriction on the input and output perturbations and derived a universal expression of MLP sensitivity applicable to antisymmetric squashing activation functions. Subsequently, Yang et al. [15] utilized the central limit theorem to develop a sensitivity expression with a more practical input distribution applicable to networks with arbitrary activation functions. Additionally, Zeng and Yeung [16] pruned the hidden layer neurons of MLP based on the relevance defined by the sensitivity and output weights, resulting in a pruned network with reduced complexity and improved generalization performance. Yang et al. [17] utilized the output sensitivity as an indicator to enhance the generalization performance of networks and select the network with superior generalization capability. Furthermore, Kapanova et al. [18] employed sensitivity to analyze network tolerance for noise, suggesting that noise may not necessarily be detrimental to the network and could potentially improve its information processing capability. In the field of SNNs, Zhang et al. [19] introduced an adaptive threshold algorithm called noise threshold, which adjusts the threshold based on whether a spike is expected during training to enhance robustness. However, this algorithm primarily focuses on increasing error tolerance and overlooks generalization performance, potentially misclassifying untrained inputs as perturbed trained inputs in practical applications.

The sensitivity analysis of SNNs presents unique challenges due to their distinct mechanisms compared to traditional neural networks. The complex neuronal dynamics used to model SNNs, with the input and output represented as discrete spike trains, makes it difficult to calculate the sensitivity through the output partial derivative or the formula for the output deviation with respect to the input perturbation. This inherent complexity leads to the inability to derive the sensitivity of SNNs using existing methods. Moreover, in contrast to traditional neural networks, SNNs involve more parameters in the feedforward process, such as the time constant, making it crucial to consider the impact of parameter perturbation on the network behavior.

In this paper, a generalized sensitivity algorithm is presented for an individual SN and subsequently for an entire SNN. The main contributions are summarized as follows:

- A definition and a computational method are presented for quantifying the sensitivity of an SNN. The sensitivity is defined as the mathematical expectation of the output deviation due to input perturbation by considering the input as a statistical variable.
- To address the inaccuracy resulting from the discrete output spike in the sensitivity derivation, an output time referred to as the desired firing time is proposed, which enables a more precise calculation of the output deviation.
- A unified method is proposed for evaluating the magnitude of temporal input perturbations.

The remaining content of this paper is as follows. Section 2 introduces an SNN model with LIF neurons and describes the notation in the network. Section 3 presents a universally applicable sensitivity definition for all types of SNs and their corresponding neural networks. Section 4 derives the specific calculation of the sensitivity from the neuron to the entire network based on the previous sensitivity definition. Section 5 conducts some experiments on the effectiveness of the sensitivity algorithm and the influence of various parameters on the sensitivity, followed by the analysis of the experimental results in Section 6. Finally, Section 7 presents a conclusion.

## 2. SNN Model and Notations

**Figure 1:**
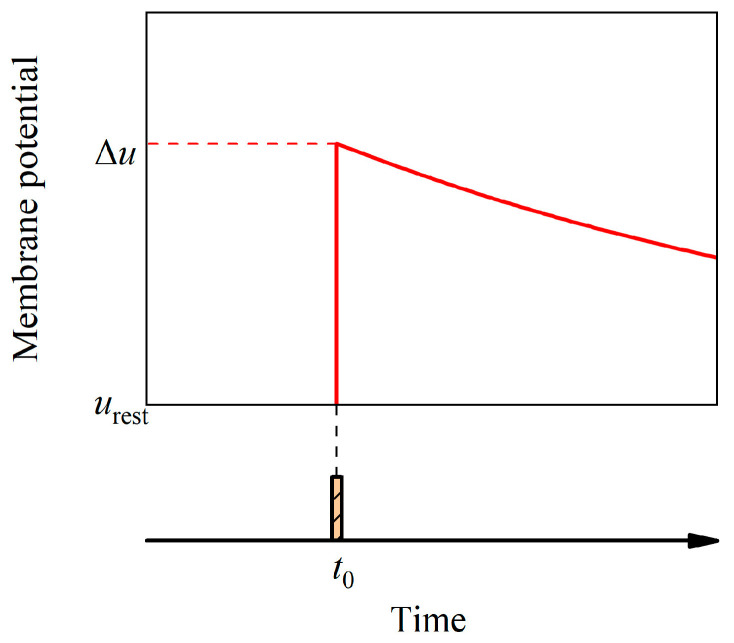
Membrane potential with a spike input. At time *t*_0_, the postsynaptic potential increases by Δ*u* as a result of receiving a spike and then exponentially decays.

Here the LIF model is chosen to describe the neuronal dynamics for the purpose of investigating the sensitivity of SNNs. For the case of a single LIF neuron, a biological input can be regarded as a short current pulse that is injected into the neuron [20]. The membrane potential *u*(*t*) is described by

(1) $$\tau \frac{du}{dt}=-\left[u\left(t\right)-u_{\mathrm{rest}}\right]+RI\left(t\right),$$

where *τ* = *RC* is the time constant, *u*_rest_ is the resting membrane potential, *R* is the membrane resistance, and *I*(*t*) is the input current. Every time an input spike arrives, the membrane potential is increased by Δ*u*. Subsequently, the membrane potential is

(2) $$u(t)-u_{\mathrm{rest}}=\exp(-\frac{t-t_{0}}{\tau })\Delta u$$

in the absence of any further input. The effect of an input spike on the membrane potential is illustrated in Figure 1. In the presence of multiple input currents, *I*(*t*′) represents the total input current over the time interval (−∞, *t*). The membrane potential continues to increase and can be expressed as

(3) $$u\left(t\right)-u_{\mathrm{rest}}=\frac{R}{\tau }\int_{-\infty }^{t}\exp\left(-\frac{t-t^{\prime }}{\tau }\right)I\left(t^{\prime }\right)dt^{\prime },$$

where the *i*-th input is *I_i_*(*t*′) = *q_i_δ*(*t*′ − *t_i_*), and *δ*(*t*′ − *t_i_*) is the Dirac delta function. The membrane potential can be further calculated as

(4) $$u\left(t\right)-u_{\mathrm{rest}}=\frac{R}{\tau }\sum_{i}q_{i}\exp\left(-\frac{t-t_{i}}{\tau }\right),$$

where *q_i_* and *t_i_* are the charge and time of the *i*-th input.

The temporal coding method is employed for information encoding. The preference for temporal coding is based on its biological plausibility, which allows for a more precise definition of sensitivity. In the temporal coding method, time serves as the carrier of information, with the spike time serving as either the input or output for single neurons [21,22].

To simplify the subsequent definition of the sensitivity of layers and networks, the notations for an SNN are introduced. In an SNN with *L* layers, layer *l* (0≤l≤L) consists of *n^l^* neurons, with *n*^0^ representing the input layer. The temporal-coded input spike for neuron *i* $\left(1\leq i\leq n^{l}\right)$ in layer *l* is denoted as $T^{l}={\left(t_{1}^{l},\ldots ,t_{n^{l-1}}^{l}\right)}^{T}$, where each element corresponds to the output from the previous layer. The synaptic weight for each neuron in this layer is denoted as $W_{i}^{l}={\left(w_{i1}^{l},\ldots ,w_{in^{l-1}}^{l}\right)}^{T}$, and the perturbation is represented as $\Delta T^{l}={\left(\Delta t_{1}^{l},\ldots ,\Delta t_{n^{l-1}}^{l}\right)}^{T}$. Each element in Δ*T^l^* corresponds to the perturbation of each element in *T^l^*, and the perturbed input is denoted as ${T^{l}}^{\prime }={\left(t_{1}^{l}+\Delta t_{1}^{l},\ldots ,t_{n^{l-1}}^{l}+\Delta t_{n^{l-1}}^{l}\right)}^{T}$. For a given layer *l* in the SNN, the input is *T^l^*, and the set of weights for that layer is represented as $W^{l}={\left(W_{1}^{l},\ldots ,W_{n^{l}}^{l}\right)}^{T}$. Throughout the entire SNN, the input is denoted as *T*^1^, and the weight is represented as $W=W^{1}\cup \ldots \cup W^{L}$.

## 3. Definitions of Sensitivity

In an SNN, the sensitivity to the input perturbation is manifested in the disparity between the perturbed and unperturbed output spike trains. This section defines the sensitivity of the SNN using a bottom–up approach. Initially, it analyzes the impact of the input perturbation on the postsynaptic neuron and provides the definition of the output sensitivity for an individual neuron. Subsequently, it establishes the definitions of the sensitivity for both individual layers and the entire network based on the sensitivity for a single neuron. The sensitivity outlined here represents, in a statistical sense, the output deviation of the SNN attributable to input deviations.

### 3.1. Definition of Sensitivity for an Individual Neuron

For an individual neuron, the most appropriate approach to defining the sensitivity is to consider the output time difference when receiving perturbed and unperturbed inputs. Despite the gradual feeding of the input spike train to the neuron over time, the information conveyed by a spike is reflected at its specific time. Thus, the spike time is utilized as an input or output in the computation. The output difference is represented as Δ*y* = *y*′ − *y*, where *y*′ is the perturbed output and *y* is the original output.

Unlike traditional neural networks, where input and output are connected through activation functions, the input time variation of an SN does not directly impact the output time. In the case of a single SN, the membrane potential *u* accumulates in response to an input spike train *T*, with the first spike occurring when the membrane potential reaches the threshold, determining the output time *y*. If the input is perturbed by Δ*T*, the new input becomes *T* + Δ*T*, resulting in a direct change in the membrane potential, Δ*u*. This change indirectly influences the firing time, causing the output time to be transformed to *y*′.

**Figure 2:**
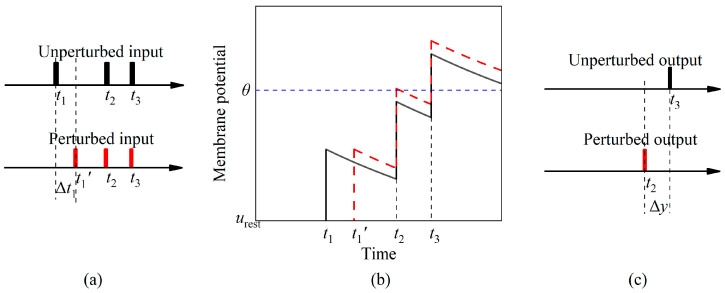
Influence of the input perturbation on the output deviation. (**a**) Input spike train. The unperturbed input spike times are represented by *t*_1_, *t*_2_, and *t*_3_ (indicated by the black lines), while the perturbed spike time is represented by *t*_1_′ (indicated by the red line). (**b**) Membrane potential after receiving the inputs. The membrane potential is shown as both red and black lines, representing the cases with and without perturbation, respectively. Without perturbation, the membrane potential crosses the threshold at time *t*_3_. However, with the perturbation, the membrane potential crosses the threshold at time *t*_2_. (**c**) Output spike train. The perturbed output time (indicated by the red line) differs from the time without perturbation (indicated by the black line).

The formation process of output deviation is specifically demonstrated in this example. It includes the input spike train, the membrane potential, and the output time, as illustrated in Figure 2. The original input is $T={\left(t_{1},\;t_{2},\;t_{3}\right)}^{T}$. Under the influence of the input spikes, the membrane potential reaches the threshold at *t*_3_, resulting in an output time of *y* = *t*_3_. When a deviation $\Delta T={\left(\Delta t_{1},\;0,\;0\right)}^{T}$ occurs in the input, the perturbed input becomes $T^{\prime }=(t_{1}+\Delta t_{1},\;t_{2},\;t_{3})$, causing the membrane potential to increase, consequently advancing the firing time. As a result, the output time becomes *y*′ = *t*_2_. In this instance, Δ*y* = *y*′ − *y* = *t*_3_ − *t*_2_ represents the output deviation for the given input and perturbation. Thus, it can be inferred that the perturbation directly affects the membrane potential, which consequently leads to possible changes in the output deviation.

When multiple spikes occur within a spike train, the sensitivity analysis should take into account the deviations of all the output spikes. The original output is $Y={\left(y_{1},\ldots ,y_{n}\right)}^{T}$, the perturbed output is $Y={\left({y_{1}}^{\prime },\ldots ,{y_{n}}^{\prime }\right)}^{T}$, and the output deviation is $\Delta Y={\left(\Delta y_{1},\ldots ,\Delta y_{n}\right)}^{T}$. In this regard, the computation of the sensitivity involves the calculation of the average deviation of these multiple outputs, which is denoted as $\Delta y=\sum_{i=1}^{n}\Delta y_{i}/n$. Our research primarily emphasizes the analysis of a single output spike, dividing the sensitivity of multiple spikes into calculations for each individual spike.

Based on the analysis conducted above, the output deviation of neuron *i* in layer *l* resulting from the input perturbation can be defined as

(5) $$\Delta y_{i}^{l}=f\left(T^{l}+\Delta T^{l},W_{i}^{l}\right)-f\left(T^{l},W_{i}^{l}\right),$$

where *f* is the mapping function between the input and output. The computation of the output deviation is dependent on the input and perturbation of an SN with fixed parameters. However, calculating $\Delta y_{i}^{l}$ for each individual input lacks significance. Instead, it is important to consider overall inputs in a statistical sense. Our definition of sensitivity aims to investigate the relationship between $\Delta y_{i}^{l}$ and Δ*T^l^* , disregarding the specific value of the input *T^l^*, by considering the mathematical expectation of $\Delta y_{i}^{l}$ when treating *T^l^* as a statistical variable. The following is the definition of sensitivity for an individual neuron.

**Definition** **1.** *For a given input deviation ΔT^l^, the sensitivity of neuron i in layer l with fixed weight $W_{i}^{l}$ is defined as the mathematical expectation of the absolute value of $\Delta y_{i}^{l}$, which is expressed as *

(6) $$s_{i}^{l}\left(\Delta T^{l}\right)=E\left(\left|\Delta y_{i}^{l}\right|\right)=E_{T^{l}}\left(\left|f\left(T^{l}+\Delta T^{l},W_{i}^{l}\right)-f\left(T^{l},W_{i}^{l}\right)\right|\right)$$

In Definition 1, $s_{i}^{l}$ remains unaffected by any particular input and serves as an evaluative measure to indicate the influence of the perturbation on the neuron.

### 3.2. Definition of Sensitivity for an Entire Network

The sensitivity of an entire SNN is determined by the sensitivity of the output layer. Thus, the definition of sensitivity for a layer is presented, followed by the definition of sensitivity for an entire network. In a fully connected SNN, each layer exhibits an all-to-all connection among its neurons. The sensitivity of a layer should be defined as the set of sensitivities of all neurons in that layer.

**Definition** **2.** *For a given input deviation ΔT^l^, the sensitivity of layer l with fixed weight W^l^ is defined as a vector containing the sensitivities of all neurons in this layer, denoted as*

(7) $$S^{l}\left(\Delta T^{l}\right)={\left(s_{1}^{l},\cdots ,s_{n^{l}}^{l}\right)}^{T}.$$

*The input perturbation of the current layer is the output sensitivity of the previous layer. Thus, when l>1, $\Delta T^{l}=S^{l-1}$*.

**Definition** **3.** *For a given input deviation ΔT^l^, the sensitivity of the entire SNN with fixed weight W is defined as the sensitivity of its output layer, which is expressed as*

(8) $$S\left(\Delta T^{1}\right)=S^{L}.$$

The sensitivity of the network can be calculated iteratively, layer by layer. It is important to note that the output of the preceding layer servers as the input of the succeeding layer. This means that the sensitivity of the preceding layer corresponds to the input perturbation of the succeeding layer, i.e., $s_{i}^{l}=\Delta t_{i}^{l+1}$ and $S^{l}=\Delta T^{l+1}$. As a result, obtaining the sensitivity layer by layer allows us to determine the sensitivity of the entire network. To facilitate the iterative computation of the sensitivity, it is crucial to define the sensitivity in a way that aligns with the time difference of the input perturbation.

## 4. Computation of Sensitivity

The sensitivity of the entire SNN is computed based on the sensitivity of individual neurons, as the sensitivity of the output layer can be determined neuron by neuron from the first layer to the last layer. Initially, the sensitivity of each neuron, represented by the expectation of the output deviation, is calculated. Subsequently, the sensitivity of the network is iteratively computed by determining the sensitivity of each layer.

### 4.1. Sensitivity of an Individual Neuron

#### 4.1.1. Desired Firing Time

**Figure 3:**
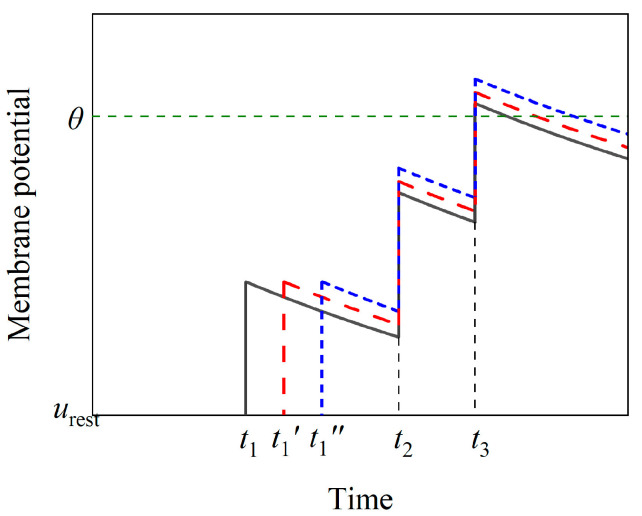
Inaccuracy of the output deviation. The variables *t*_1_, *t*_2_, and *t*_3_ represent the unperturbed inputs (black line), while *t*_1_′ (red line) and *t*_1_″ (blue line) represent the perturbed inputs. It is noted that the output deviations due to the perturbed inputs *t*_1_′ and *t*_1_″ are both Δ*y* = *t*_3_ − *t*_3_ = 0.

Inaccuracies arise when calculating the sensitivity of a neuron directly using the output time derived from the LIF model. This is due to the event-driven nature of SNNs and the discreteness of spike trains, which means the firing time of a neuron can only be a specific input time. For a given input, assume that the unperturbed firing time of the neuron is *t_m_*. If the membrane potential does not trigger a spike at any time other than *t_m_* after being perturbed, the output deviation is 0. Conversely, if the neuron fires at another input time *t_m_*′ after being perturbed, the output deviation is represented by Δ*y* = *t_m_*′ − *t_m_*. In this way, the output deviation signifies whether the output is shifted by a discrete Δ*y*. Furthermore, when the input perturbations are small, different input perturbations can result in varying potential perturbations, yet the output deviations may remain the same, being 0. Consequently, the sensitivity under different input perturbations may all be 0, as illustrated in Figure 3. The nuanced variations in the influence of input perturbation on the potential are not manifested in the output deviation. Hence, the output deviation needs to be continuous and accurate to ensure that the calculated sensitivity maintains precision.

The inaccuracy in sensitivity can be attributed to the event-driven nature of SNNs, which results in a non-continuous increase in the membrane potential over time. Ideally, a neuron is expected to emit a spike immediately when the membrane potential reaches the threshold *θ*. However, the membrane potential exceeds the threshold at the actual firing time. If the membrane potential increases continuously with the input spikes, the firing time advances.

**Figure 4:**
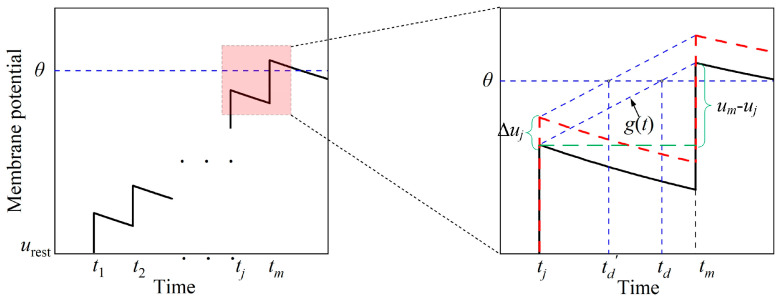
Definition of the desired firing time. The solid black line represents the original membrane potential, while the dashed red line represents the perturbed membrane potential. The variable *t_m_* is the actual firing time, *t_j_* is the moment with the highest potential before the actual firing time, *t_d_* is the desired firing time, *t_d_*′ is the desired firing time after perturbation, and the output deviation is calculated as Δ*y* = *t_d_*′ − *t_d_*.

The desired firing time is introduced to improve the accuracy of the calculation of the output deviation. It is defined as the moment when the membrane potential precisely reaches the threshold, assuming a uniform increase in the membrane potential before firing. Figure 4 illustrates the definition of the desired firing time and the difference in output deviation between using the desired firing time and the actual firing time. The function *g*(*t*) in Figure 4 represents the linear increase in membrane potential between *t_m_* and *t_j_*; *t_m_* represents the actual firing time and *t_j_* is the moment with the highest potential before the actual firing time. The desired firing time *t_d_* occurs when *g*(*t_d_*) = *θ*. If there is an input perturbation that increases the membrane potential, *g*(*t_d_*) shifts upward and the desired firing time uniformly decreases. The desired firing time changes from *t_d_* to *t_d_*′. The output deviation using the desired firing time, *t_d_*′ − *t_d_*, is more accurate than the original output deviation using the actual firing time, which is 0. By calculating sensitivity using the difference between the unperturbed and perturbed desired firing times, subtle input perturbations can more precisely influence the output time, resulting in a more accurate sensitivity. This is in contrast to merely causing changes in the membrane potential.

#### 4.1.2. Computation of Sensitivity for an Individual Neuron

To calculate the sensitivity for an individual neuron, the expression for the output deviation should first be obtained. A more precise output deviation can be achieved by using the desired firing time, as shown in Figure 4. In relation to a particular input, the absolute value of the output deviation can be calculated as

(9) $$\left|\Delta y\right|=\left|{t_{d}}^{\prime }-t_{d}\right|=\left|\frac{t_{m}-t_{j}}{u_{m}-u_{j}}\Delta u_{j}\right|,$$

where *t_m_* represents the actual firing time without perturbation, *t_j_* is the moment with the highest potential prior to the actual firing time, *u_m_* and *u_j_* are the membrane potentials corresponding to tm and *t_j_*, respectively, and Δ*u_j_* represents the difference between the perturbed potential and the unperturbed potential at *t_j_*. In accordance with Definition 1, the sensitivity of a neuron can be computed as

(10) $$s=E\left(\left|\Delta y\right|\right)=E\left(\left|\frac{t_{m}-t_{j}}{u_{m}-u_{j}}\Delta u_{j}\right|\right).$$

When the input perturbation Δ*T* > 0, it follows that *t_m_* > *t_j_* and *u_m_* > *u_j_*, resulting in Δ*y* > 0. The sensitivity can thus be simplified to

(11) $$s=E\left(\Delta y\right)=E\left(\frac{t_{m}-t_{j}}{u_{m}-u_{j}}\Delta u_{j}\right).$$

In order to continue the calculation of the sensitivity, it is necessary to derive the expressions for the membrane potential *u* and the potential difference Δ*u*. The membrane potential *u* is presented in (4), where $\frac{R}{\tau }\sum_{i}q_{i}$ represents the weight of the *i*-th input. The expression for the membrane potential is

(12) $$u=\sum_{i}w_{i}\exp\left(-\frac{t-t_{i}}{\tau }\right),$$

where Δ*u* can be considered as the membrane potential difference obtained when *T* and *T* + Δ*T* are used as inputs. The mathematical expression for Δ*u* is given by

(13) $$\Delta u=u^{\prime }-u=\sum_{i}w_{i}\exp\left(-\frac{t-\left(t_{i}+\Delta t_{i}\right)}{\tau }\right)-\sum_{i}w_{i}\exp\left(-\frac{t-t_{i}}{\tau }\right).$$

According to (12) and (13), the expression within the expectation in (10) can be derived as

(14) $$\frac{\left(t_{m}-t_{j}\right)\Delta u_{j}}{u_{m}-u_{j}}=\left(t_{m}-t_{j}\right)\frac{\sum_{i=1}^{j}w_{i}\exp\left(-\frac{t_{j}-\left(t_{i}+\Delta t_{i}\right)}{\tau }\right)-\sum_{i=1}^{j}w_{i}\exp\left(-\frac{t_{j}-t_{i}}{\tau }\right)}{\sum_{i=1}^{m}w_{i}\exp\left(-\frac{t_{m}-t_{i}}{\tau }\right)-\sum_{i=1}^{j}w_{i}\exp\left(-\frac{t_{j}-t_{i}}{\tau }\right)}.$$

According to the principle of the mathematical expectation of multivariate random variables, the expectation is given by the integral of the product of the probability density function and the mapping function. Considering all inputs *T* = (*t*_1_, *t*_2_, …, *t_n_*) as statistical variables, the sensitivity in (10) can be expressed as

(15) $$s=E(\Delta y)=E\left(\frac{\left(t_{m}-t_{j}\right)\Delta u_{j}}{u_{m}-u_{j}}\right)=\int_{\Omega_{T}}\varphi \left(T\right)\left(t_{m}-t_{j}\right)\frac{\Delta u_{j}}{u_{m}-u_{j}}d\Omega_{T},$$

where *φ*(*T*) is the density function and Ω*_T_* is the region spanned by input vector *T*. If (*X*_1_, *X*_2_, …, *X_n_*) are independent and identically distributed as uniform (0, 1), ordering these *n* random variables produces the ordered sequence (*X*_(1)_, …, *X*_(*n*)_), where the distribution of *X*_(*k*)_ is Beta(*k*, *n* − *k*+1). Given that the input *T* = (*t*_1_, *t*_2_, …, *t_n_*) is uniformly distributed, the input in (15) is normalized and ordered to yield the sequence *T*/*λ*. Here, *X* = *T*/*λ* represents the quotient obtained by dividing the input by the number of time steps, and it is uniformly distributed on [0, 1]. Let *X_k_* denote the *k*-th order statistic in *T*/*λ*, and then *X_k_* follows a Beta(*k*, *n* − *k*+1) distribution. According to the definition of the Beta distribution, the density function is given by

(16) $$\varphi_{k}\left(x_{k}\right)=\frac{x_{k}^{k-1}{\left(1-x_{k}\right)}^{n-k}}{B\left(k,n-k+1\right)}=\frac{x_{k}^{k-1}{\left(1-x_{k}\right)}^{n-k}}{\int_{0}^{1}v^{k-1}{\left(1-v\right)}^{n-k}dv}.$$

Substituting (14) and (16) into (15), the sensitivity expression can be calculated as

(17) $$s=\int_{\Omega_{X}}\prod_{i=1}^{m}\left(\varphi_{i}\left(x_{i}\right)\right)\frac{\left(\lambda x_{m}-\lambda x_{j}\right)\left(\sum_{i=1}^{j}w_{i}\exp\left(-\frac{\lambda x_{j}-\left(\lambda x_{i}+\Delta t_{i}\right)}{\tau }\right)-\sum_{i=1}^{j}w_{i}\exp\left(-\frac{\lambda x_{j}-\lambda x_{i}}{\tau }\right)\right)}{\sum_{i=1}^{m}w_{i}\exp\left(-\frac{\lambda x_{m}-\lambda x_{i}}{\tau }\right)-\sum_{i=1}^{j}w_{i}\exp\left(-\frac{\lambda x_{j}-\lambda x_{i}}{\tau }\right)}d\Omega_{X}.$$

Simplified calculations can be achieved by employing the following approximation method:

(18) $$\frac{1}{1+x}\approx \sum_{r=0}^{p}{\left(-1\right)}^{r}x^{r}\mathrm{for}\;\left|x\right|<1,\;\mathrm{where}\;1\leq p<+\infty ,$$

(19) $$\exp(x)\approx \sum_{h=0}^{q}\frac{x^{h}}{h!},\;\mathrm{where}\;1\leq q<+\infty .$$

After approximating, simplifying, and integrating, the sensitivity presented in (17) can be derived as

(20) $$\begin{array}{c} s=E\left(\frac{\left(t_{m}-t_{j}\right)\Delta u_{j}}{u_{m}-u_{j}}\right)=\left(\frac{\lambda \left(m-j\right)}{n+1}\right)\left(\sum_{i=1}^{j-1}w_{i}\left(\frac{\Delta t_{i}}{\tau }+\frac{\Delta t_{i}^{2}}{2\tau^{2}}+\frac{\lambda \Delta t_{i}i}{\tau^{2}\left(n+1\right)}\right)\right)\left(1-\frac{\lambda j}{\tau \left(n+1\right)}+\frac{\lambda^{2}\left(j+1\right)j}{2\tau^{2}\left(n+2\right)\left(n+1\right)}\right)\\ \left(\frac{1}{w_{m}}-\frac{1}{w_{m}^{2}}\sum_{i=j+1}^{m-1}w_{i}\left(1+\frac{\lambda i}{\tau \left(n+1\right)}+\frac{\lambda^{2}\left(i+1\right)i}{2\tau^{2}\left(n+2\right)\left(n+1\right)}\right)\right)\left(1-\frac{\lambda m}{\tau \left(n+1\right)}+\frac{\lambda^{2}\left(m+1\right)m}{2\tau^{2}\left(n+2\right)\left(n+1\right)}\right) \end{array}.$$

#### 4.1.3. Moment with Highest Potential Before Actual Firing Time

The primary rationale for utilizing *u_j_* as opposed to *u_m_*_−1_ to determine the sensitivity of a neuron is that *u_m_*_-1_ may not represent the maximum value of membrane potential prior to the firing time *t_m_*. When calculating the sensitivity, it is necessary to determine the value of *j* by considering the time *t_j_* at which firing is most easily triggered after perturbation. For a neuron with fixed weights, the increase in membrane potential caused by any input is known. Hence, *j* can be determined by finding the maximum expectation of the membrane potential before *t_m_*. According to (12), the expectation of the membrane potential before *t_m_* is

(21) $$E\left(u_{j}\right)=\int_{\Omega_{T}}\varphi \left(T\right)\sum_{i=1}^{j}w_{i}\exp\left(-\frac{t_{j}-t_{i}}{\tau }\right)d\Omega_{T}.$$

By utilizing an approximation method grounded in (19), one can proceed with the computation of (21) as

(22) $$E\left(u_{j}\right)=\left(\sum_{i=1}^{j-1}w_{i}\left(1+\frac{\lambda i}{\tau \left(n+1\right)}+\frac{\lambda^{2}\left(i+1\right)i}{2\tau^{2}\left(n+2\right)\left(n+1\right)}\right)\right)\left(1-\frac{\lambda j}{\tau \left(n+1\right)}+\frac{\lambda^{2}\left(j+1\right)j}{2\tau^{2}\left(n+2\right)\left(n+1\right)}\right)+w_{j}.$$

In summary, it is essential to determine the moment with the highest potential before the firing time prior to calculating sensitivity. This can be achieved by evaluating the expectation of the membrane potential with perturbation using (22). Then, the maximum potential, represented as *u_j_*, is determined. The corresponding time *t_j_* is the result.

#### 4.1.4. Measure of Input Perturbation

A unified quantitative measure is required to determine the magnitude of input perturbation Δ*T*. An input perturbation can be represented by a sequence of perturbations corresponding to input times, denoted as $\Delta T={\left(\Delta t_{1},\ldots ,\Delta t_{n}\right)}^{T}$. It is important to note that different inputs with the same deviation can lead to varying output deviations. Hence, comparing the magnitude of input perturbations solely based on the value of any input deviation Δ*t_i_* is not feasible.

The expectation of the potential difference quantifies the deviation resulting from a specific perturbation with respect to all inputs. It is chosen as the measure of the magnitude of input perturbations. The potential difference after receiving $T={\left(t_{1},\ldots ,t_{m}\right)}^{T}$ is

(23) $$\Delta u_{m}=\sum_{i=1}^{m}w_{i}\exp\left(-\frac{t_{m}-\left(t_{i}+\Delta t_{i}\right)}{\tau }\right)-\sum_{i=1}^{m}w_{i}\exp\left(-\frac{t_{m}-t_{i}}{\tau }\right).$$

The expectation is calculated using the approximate method outlined in (19) as

(24) $$E\left(\Delta u_{m}\right)=\left(\sum_{i=1}^{m-1}w_{i}\left(\frac{\Delta t_{i}}{\tau }+\frac{\Delta t_{i}^{2}}{2\tau^{2}}+\frac{\lambda \Delta t_{i}i}{\tau^{2}\left(n+1\right)}\right)\right)\left(1-\frac{\lambda m}{\tau \left(n+1\right)}+\frac{\lambda^{2}\left(m+1\right)m}{2\tau^{2}\left(n+2\right)\left(n+1\right)}\right).$$

In conclusion, the magnitude of any input perturbation can be quantified by *E*(Δ*u_m_*).

### 4.2. Computation of Sensitivity for an Entire SNN

The sensitivity of an entire SNN can be calculated iteratively, layer by layer. The input perturbation of each layer Δ*T^l^* is determined by the output sensitivity of the previous layer *S^l−^*^1^, as defined in Definition 2. When *l* = 1, the input perturbation is represented by Δ*T*^1^, and the sensitivity of layer 1 is computed by evaluating the sensitivity of each neuron in that layer. For *l* ≥ 2, the input perturbation is represented by Δ*T^l^* = *S^l−^*^1^, and the sensitivity of layer *l* can be computed as *S^l^*(*S^l^*^−1^). Then, the sensitivity of the entire SNN, i.e., the sensitivity of layer *L*, is obtained iteratively. Algorithm 1 calculates the sensitivity of an entire SNN, with the input perturbation Δ*T*^l^ = *S^0^*.
**Algorithm 1: SNN_Sensitivity(Δ*T*^1^):**  For *l* = 1 to *L* step 1
    For *i* = 1 to *n^l^* step 1
      Compute *j* by using (22)
      $\mathrm{Compute}\;s_{i}^{l}(S^{l-1})$ by using (20)
    $\mathrm{Compute}\;S^{l}=\Delta T^{l+1}=(s_{1}^{l},\ldots ,s_{n^{l}}^{l})$
  Compute *S* = *S^L^*

## 5. Experiment

A series of experiments were carried out to validate the proposed algorithms. Initially, the effectiveness of the sensitivity algorithm for individual neurons is proven by comparing the theoretical and simulation results with varying parameters. Subsequently, the sensitivity algorithm for SNNs with varying weights is validated, and an investigation is conducted to assess the impact of the input dimension on the sensitivity.

The hardware and software platforms used for all experiments are as follows: CPU, Intel i7 10700K; GPU, NVIDIA 3070; programming language, Python 3.8.18; development framework, PyTorch 2.1.2; and operating system, Windows 10.

### 5.1. Sensitivity of Individual Neurons

Several experiments were performed to compare the theoretical and simulation results of the sensitivity with various parameters. The primary objective is to validate the effectiveness of the computational approach to sensitivity and examine the influences of varying parameters on the sensitivity. The subsequent four experiments aim to investigate the relationship between the input perturbation and the sensitivity, considering variations in the input perturbation, the time constant, the number of time steps, and the input dimension.

#### 5.1.1. Input Perturbation

The first experiment aims to validate the plausibility of the theoretical calculation scheme for determining the sensitivity of an individual neuron. The parameters of the neuron are listed in Table 1. Specifically, the sensitivities of a neuron with 20 input synapses are calculated by introducing various input perturbations. The parameters, such as the number of time steps and the time constant, remain constant, with the exception of the weights. Three distinct sets of weights labeled as *W*_1,_ *W*_2_, and *W*_3_ are randomly generated with a uniform distribution. The theoretical results are calculated using (20), while the simulation results are derived from the average output deviation of 1 million input samples. The average computation time for the simulation results is about 41 min. All parameters and the input perturbations used in the calculation of both the theoretical and simulation sensitivities share identical values.

**Figure 5:**
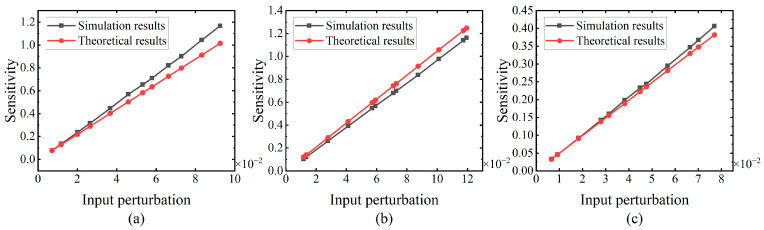
Trend of the sensitivity variation in response to the input perturbations under the three distinct weights. The number of the input synapses is 20, the number of time steps are 64, and the time constant is 50 ms. The simulation results are depicted by the black line, while the theoretical results are represented by the red line. The weights in the figure are (**a**) *W*_1_, (**b**) *W*_2_, and (**c**) *W*_3_.

The sensitivities obtained are illustrated in Figure 5, with the magnitude of the input perturbations being represented by the expectation of the potential difference. Figure 5 shows that, under the three different weights, both the theoretical and simulation results exhibit an upward trend in response to the increasing input perturbation. This implies that greater input perturbation results in higher sensitivity for an SN with fixed parameters. Furthermore, the theoretical results are in reasonable agreement with the simulation results, showing consistent trends. In conclusion, the effectiveness of the theoretical sensitivity calculation with varying weights is demonstrated.

#### 5.1.2. Time Constant

The second experiment aims to investigate the sensitivity variation with respect to the time constant. All parameters, with the exception of the time constant, are held constant and are listed in Table 2. Three perturbations, labeled as Δ*T*_1_, Δ*T*_2_, and Δ*T*_3_ in ascending order of magnitude, are utilized as input perturbations. A total of 24 different time constants ranging from 39 ms to 62 ms with a 1 ms increment are employed in the experiment under the three different perturbations. The average computation time for the simulation results is about 40 min.

**Figure 6:**
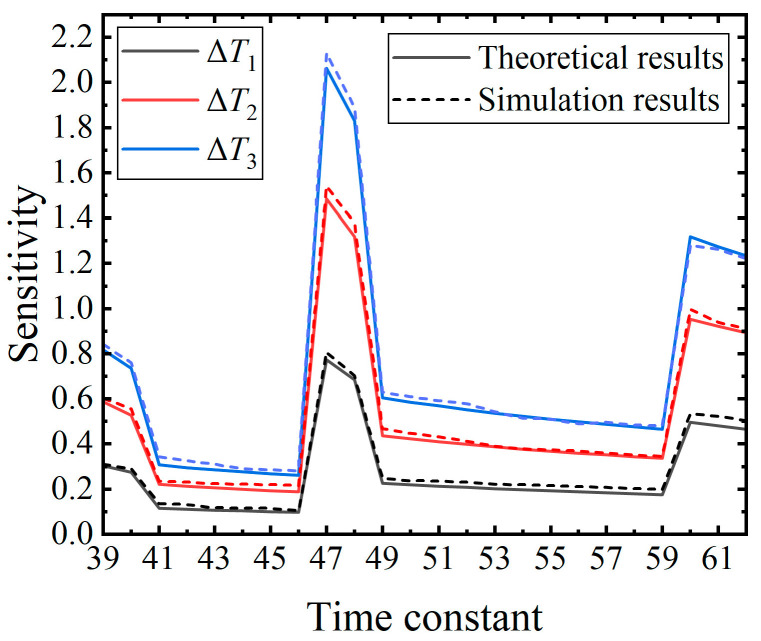
Trend of the sensitivity variation in response to the time constants under the three distinct perturbations. The number of time steps and the number of the input synapses are consistent with those of the first experiment. The weights of the synapses are randomly generated. The sensitivity corresponding to Δ*T*_1_, Δ*T*_2_, and Δ*T*_3_ is indicated by the black line, red line, and blue line, respectively.

As depicted in Figure 6, the sensitivity exhibits a sudden increase at time constants of 39 ms, 47 ms, and 60 ms, followed by a gradual decrease as the time constant further increases. The findings indicate a piecewise decreasing trend in sensitivity with respect to the time constant. By referring to the definition of the membrane potential difference expressed in (13), the impact of a specific input perturbation on the membrane potential diminishes as the time constant increases, leading to reduced attenuation of the membrane potential. It can be inferred that the sensitivity declines with an increasing time constant. Therefore, within a variation range of the time constant (e.g., from 47 ms to 60 ms), the experimental result regarding sensitivity aligns with the inference from the definition. The sudden increase in the sensitivity can be attributed to the fact that the increased time constant induces a reduction in the membrane potential, consequently altering the firing time. The desired firing time changes at time constants of 39 ms, 47 ms, and 60 ms in this experiment. As a result, the sensitivity abruptly increases at specific moments and then gradually decreases.

Figure 6 also reveals that the sensitivity obtained from the smallest perturbation Δ*T*_1_ (black line) is smaller than the sensitivity obtained from the medium perturbation Δ*T*_2_ (red line) at any time constant. Similarly, the sensitivity obtained from Δ*T*_2_ is smaller than the sensitivity obtained from the largest perturbation Δ*T*_3_ (blue line). This phenomenon confirms the findings of the first experiment from a different perspective, demonstrating that greater perturbations yield higher sensitivity, a relationship that holds true at various time constants.

#### 5.1.3. Number of Time Steps

The third experiment aims to investigate the impact of the number of time steps on the sensitivity. In this experiment, all parameters other than the number of time steps are kept fixed. Except for the parameters in Table 3, all others are identical to those used in the second experiment. The experiment was conducted with 12 different numbers of time steps ranging from 55 to 66 under the three input perturbations, and the corresponding theoretical sensitivities were obtained. The simulation results were obtained using identical parameters. The average computation time for the simulation results is about 36 min.

**Figure 7:**
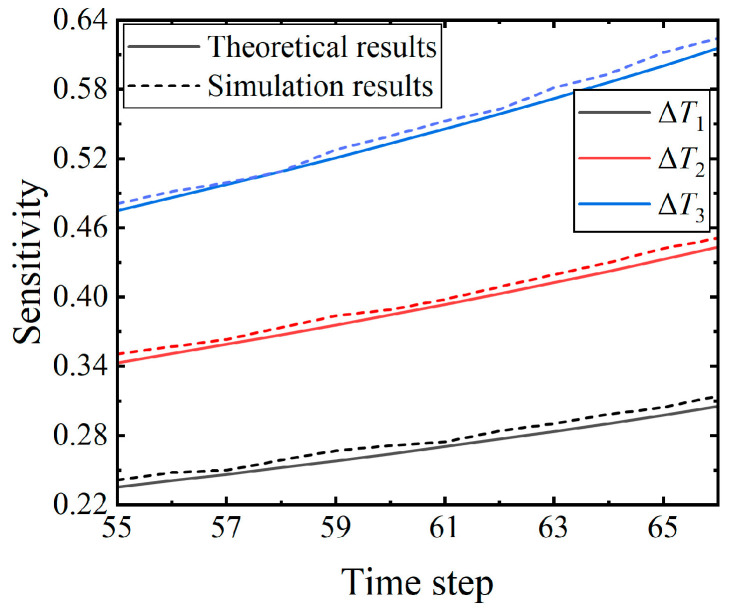
Trend of the sensitivity variation in response to the number of time steps under the three distinct perturbations. The number of input synapses and the values of the weights are consistent with those of the second experiment. The time constant is 50 ms.

The theoretical results, depicted in Figure 7, imply that the sensitivity increases with the number of time steps under the three different input perturbations. From the perspective of the neuronal dynamics theory, with the fixed number of input synapses, larger time steps lead to sparser inputs, resulting in a larger average output deviation and consequently increased sensitivity. Thus, the theoretical results in this experiment are consistent with the neuronal dynamics theory. Furthermore, this experiment illustrates that greater perturbation leads to higher sensitivity, holding true at various time steps, which supports the result of the first experiment.

#### 5.1.4. Input Dimension

The objective of the fourth experiment is to examine the impact of the input dimension on the sensitivity. All parameters, except the input dimension, are held constant. Except for the parameters in Table 4, all others are identical to those used in the second experiment. To prevent errors resulting from insufficient firing due to a low number of inputs, the input dimension is set to a total of 11 integers ranging from 30 to 40. All parameters used in the calculation of both the theoretical and simulation results share identical values. The average computation time for the simulation results is about 112 min.

**Figure 8:**
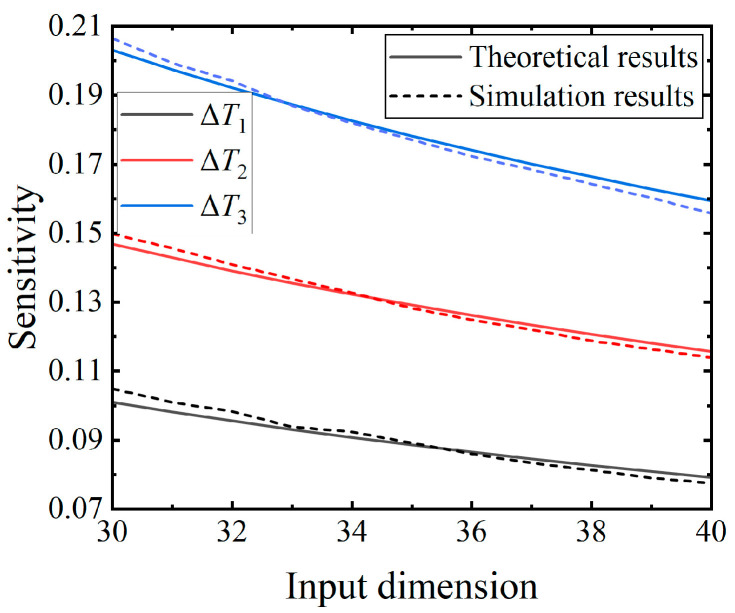
Trend of the sensitivity variation in response to the input dimensions under the three distinct perturbations. The number of time steps is 128, and the time constant is 80 ms. The weights of the input synapses are consistent with those of the second experiment.

The results depicted in Figure 8 illustrate a gradual decrease in sensitivity as the input dimension increases under the three distinct input perturbations. In line with the neuronal dynamics theory, the expansion of the input dimension leads to a more compact spike representation across the entire input spike train, resulting in a smaller perturbed output difference and, consequently, reduced sensitivity. As a result, the results corroborate the theoretical inference and demonstrate the accurate calculation of the proposed sensitivity formula across a range of input dimensions.

### 5.2. Sensitivity of Entire SNNs

Two experiments are conducted to investigate the characteristic of sensitivity for entire SNNs. Random weights ranging from 0 to 1 are assigned in both experiments. The number of time steps for each neuron is 64 and the time constant is 50 ms.

**Figure 9:**
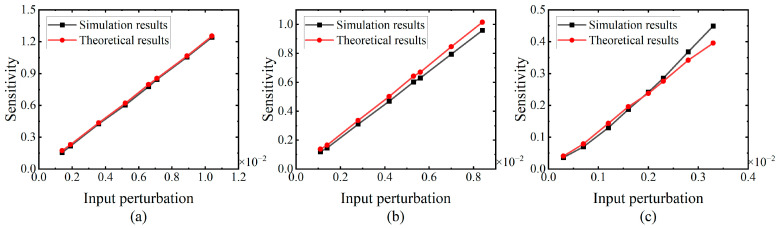
Trend of the sensitivity variation for the SNNs in response to the input perturbations. The black line represents the simulation results, while the red line represents the theoretical results. The weights in the figure are (**a**) *W*_4_, (**b**) *W*_5_, and (**c**) *W*_6_.

The fifth experiment is designed to validate the effectiveness of the proposed algorithm SNN_Sensitivity (Δ*T*^1^). Specifically, the SNN structure is 20-5-1, comprising 20 neurons in the input layer, 5 neurons in the hidden layer, and 1 neuron in the output layer. Three sets of network weights, *W*_4_, *W*_5_, and *W*_6_, are randomly chosen for the experiment. The theoretical results are computed using the algorithm SNN_Sensitivity (Δ*T*^1^), while the simulation results are determined by averaging the output deviations from the network using 1 million uniformly distributed random spike trains as inputs. The average computation time for the simulation results is about 127 min. Figure 9 presents the experimental result, illustrating that both the theoretical and simulation sensitivities increase as input perturbations amplify under the three weight sets. The alignment between the outcomes of the entire SNNs and the individual SNs implies that the observed trend applies universally to SNNs with varying parameters. Moreover, the experimental results indicate that the theoretical sensitivity derived from the algorithm SNN_Sensitivity (Δ*T*^1^) reasonably agrees with the simulation sensitivity. Consequently, the proposed algorithm demonstrates practical significance and reasonability.

**Figure 10:**
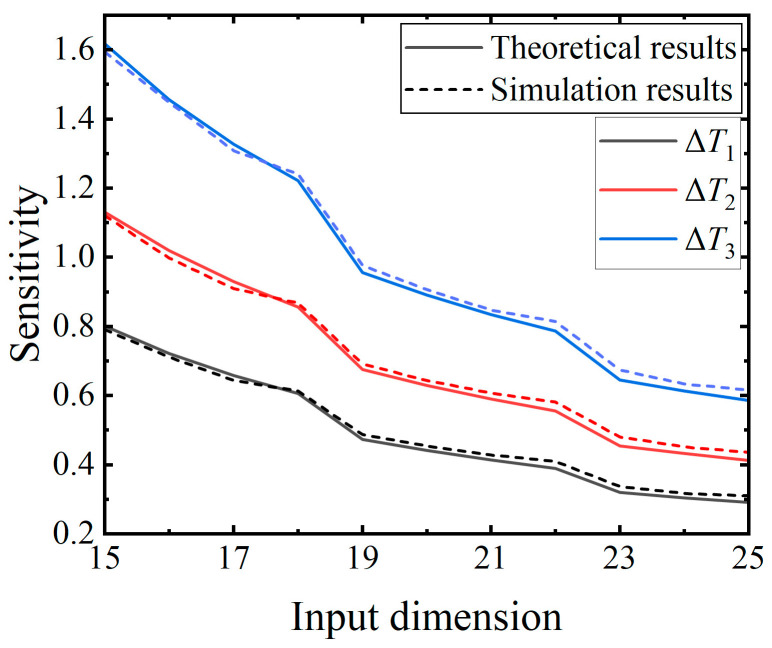
Trend of the sensitivity variation for the SNNs in response to the input dimensions. The three distinct sensitivity curves represent the results with Δ*T*_1_ (black line), Δ*T*_2_ (red line), and Δ*T*_3_ (blue line).

The sixth experiment seeks to explore the influence of the input dimension on SNN sensitivity using a network structure of *n*^0^-5-1. To mitigate errors resulting from insufficient inputs that cannot trigger firing, a range of 15 to 25 is selected for the input dimension, comprising 11 numbers. Three input perturbations, consistent with those of the second experiment, are utilized to obtain the theoretical results and the simulation results. The average computation time for the simulation results is about 131 min. Figure 10 illustrates that the sensitivity diminishes as the input dimension increases. This result aligns with the response of individual SNs to input dimensions, suggesting its applicability to SNNs of various input dimensions.

## 6. Discussion

This paper proposes a methodology for quantifying the sensitivity of temporal-coded SNs and presents the sensitivity algorithm SNN_Sensitivity(Δ*T*_1_) for SNNs. Summarizing the key findings, our experimental results reveal a strong consistency between the theoretical and simulation outcomes across various input perturbations and weight configurations. This confirms the reliability and effectiveness of the proposed sensitivity schemes for SNs and SNNs. Furthermore, the experiments also illustrate the influence of various parameters and network structures on SNN sensitivity, providing a better evaluation of networks with differing parameters.

The empirical investigations into the influence of parameters on sensitivity unveil several significant insights. Across all experiments conducted, there is consistent evidence indicating that sensitivity can exhibit variations even when individual parameters are held at the same value. Although individual parameters do exert an influence on sensitivity, they are not the sole determinants. This implication points to the limitation of relying on a single parameter as a direct measure of the significance of an SN within an SNN. Conversely, the sensitivity, with its capacity to capture nuanced correlations, emerges as a pivotal metric for assessing the contributory role of an SN in an SNN. It also occupies a critical position in the development and refinement of pruning algorithms. Furthermore, the fourth experiment reveals that increasing the input dimension results in decreased sensitivity, leading to poorer generalization performance of the network. This phenomenon can be attributed to the perception of an untrained input as a perturbed variant of a trained input, making it easier for a low-sensitive network to misclassify this new input as a known trained input.

Despite numerous investigations, a definitive conclusion regarding the influence of the time constant on sensitivity remains elusive. The experimental results indicate that sensitivity undergoes a pronounced surge at certain specific values of the time constant, followed by a gradual decline. A plausible explanation for this observed phenomenon is that as the time constant increases, not only does the impact of the perturbation on the membrane potential diminish, but the effect of the input on the membrane potential also diminishes, leading to an overall decrease in the membrane potential. If the alteration in the time constant induces a shift in the firing time, it can be observed from the definition of the membrane potential difference that the impact of the input perturbation suddenly increases. To summarize, the sensitivity exhibits a sudden surge and a subsequent reduction commences.

During the validation process of the proposed sensitivity algorithm, although the theoretical and simulation results are numerically close, there are still some unavoidable errors. The first reason for the errors is the employment of the approximate methods in the derivation of the theoretical sensitivity, specifically (18) and (19), where larger values of *p* and *q* result in smaller error. Nonetheless, *p* is set to 1 and *q* is set to 3 to simplify the subsequent calculations in the experiments. The second reason is the use of the desired firing time for the sensitivity calculation. Although this approach offers an improvement in accuracy when compared to utilizing the actual firing time, it still bears a small margin of error.

The proposed sensitivity calculation in (20) is derived under the assumption that the input spike train follows a uniform distribution. When the input does not follow a uniform distribution, the probability density function in (16) is no longer valid, and using (20) to calculate sensitivity may lead to significant errors. Therefore, it is necessary to analyze the actual input encountered, determine its probability density function, and substitute it into (15) to obtain a more accurate sensitivity formula. In summary, the sensitivity definition and the derivation up to (16) are general. If the specific input distribution can be modeled, a sensitivity calculation more suitable for real-time implementation can be derived.

The proposed sensitivity algorithm for SNNs will offer various advantages in practical applications. This is attributed to its ability to reflect the output deviation with respect to overall inputs, thereby characterizing the properties of a trained SNN. By considering the output deviation as an error with respect to the input perturbation, the sensitivity can be used to assess the error tolerance of an SNN. Alternatively, when the output deviation is perceived as an increment, the sensitivity serves as an indicator for assessing the generalization performance of an SNN. By utilizing sensitivity as a measure of correlation between neurons, it can also be applied as a criterion for network structure pruning.

The proposed sensitivity algorithm is specifically tailored for temporal-coded SNNs, analyzing the impact of subtle temporal perturbations. Other coding schemes, such as rate coding, could theoretically undergo a similar method for sensitivity calculation. Nonetheless, evaluating the sensitivity of SNNs with non-time-focused coding schemes would be meaningless, as they emphasize frequency changes instead of temporal changes. Therefore, further research is essential to develop a more universal SNN sensitivity assessment algorithm applicable to various coding schemes. Moreover, sensitivity algorithms for more complex convolutional and recurrent SNNs, beyond the fully connected SNNs used in this study, require further investigation. Finally, the experimental results assume uniformly distributed inputs, leading to general outcomes. However, in practical applications, using appropriate input distributions is necessary to obtain more accurate sensitivities.

## 7. Conclusions

This paper introduces a generalized sensitivity algorithm for fully connected SNNs. Our approach utilizes the output deviation as a quantifiable metric to assess the sensitivity in response to the input perturbation. To overcome the error caused by the discrete nature of the spike train, the concept of “the desired firing time” as a foundational element is introduced in our sensitivity computations. Subsequently, the computation of the sensitivity for an individual LIF neuron is proposed by calculating the expectation of the output deviation. Building upon this foundation, an iterative algorithm is designed for computing the sensitivity of fully connected SNNs. The experimental results underscore the effectiveness of the proposed sensitivity algorithm, exhibiting strong alignment with simulation outcomes. The conclusions regarding the impact of parameters on SNN sensitivity will significantly contribute to assessing the error tolerance and generalization capability. The proposed sensitivity analysis of SNNs offers a potent method of accurately assessing network behavior, thereby guiding their design and optimal selection.

## Figures and Tables

**Table 1:** Parameter settings of Experiment 1.

Parameter	Description	Value
Input dimension	Number of elements in the input *T*	20
Time constant	The parameter of the quantity that determines the exponential decay	50
Time steps	Number of time steps	64
Input perturbation	Range of input perturbation	[0, 0.12]
Weight	Weight of the neuron	*W*_1,_ *W*_2_, and *W*_3_

**Table 2:** Parameter settings of Experiment 2.

Parameter	Description	Value
Input dimension	Number of elements in the input *T*	20
Time constant	The parameter of the quantity that determines the exponential decay	[39, 62]
Time steps	Number of time steps	64
Input perturbation	Perturbation of input *T*	Δ*T*_1_, Δ*T*_2_, and Δ*T*_3_
Weight	Weight of the neuron	*W*_1_

**Table 3:** Parameter settings of Experiment 3.

Parameter	Description	Value
Time constant	The parameter of the quantity that determines the exponential decay	50
Time steps	Number of time steps	[55, 66]

**Table 4:** Parameter settings of Experiment 4.

Parameter	Description	Value
Input dimension	Number of elements in the input *T*	[30, 40]
Time constant	The parameter of the quantity that determines the exponential decay	80
Time steps	Number of time steps	128

## Data Availability

The data presented in this study are available on request from the corresponding author due to the absence of special open data repositories and the storage of data by the authors of the study.
